# Muscle Cramps and Neuropathies in Patients with Allogeneic Hematopoietic Stem Cell Transplantation and Graft-versus-Host Disease

**DOI:** 10.1371/journal.pone.0044922

**Published:** 2012-09-17

**Authors:** Peter D. Kraus, Daniel Wolff, Oliver Grauer, Klemens Angstwurm, Sven Jarius, Klaus P. Wandinger, Ernst Holler, Wilhelm Schulte-Mattler, Ingo Kleiter

**Affiliations:** 1 Department of Neurology, University Medical Centre Regensburg, Regensburg, Germany; 2 Department of Hematology and Clinical Oncology, University Medical Centre Regensburg, Regensburg, Germany; 3 Division of Molecular Neuroimmunology, Department of Neurology, University of Heidelberg, Heidelberg, Germany; 4 Institute for Experimental Neuroimmunology, affiliated to Euroimmun, Lübeck, Germany; 5 Department of Neurology, St. Josef-Hospital, Ruhr-University Bochum, Bochum, Germany; Julius-Maximilians-Universität Würzburg, Germany

## Abstract

**Objective:**

Graft-versus-host disease (GVHD) is an immune-mediated multisystemic disorder and the leading cause of morbidity after allogeneic hematopoietic stem cell transplantation. Peripheral nervous system manifestations of GVHD are rare but often disabling. Whereas immune-mediated neuropathies are an established feature of GVHD, muscle cramps are not well characterized.

**Methods:**

In a single-centre retrospective cohort we studied 27 patients (age 23 to 69 years) with GVHD (acute n = 6, chronic n = 21) who complained of symptoms suggestive of peripheral nervous system complications. Clinical, laboratory and neurophysiological findings were evaluated by descriptive statistics and regression analysis to detect factors associated with muscle cramps. Patient’s sera were examined for anti-neuronal antibodies.

**Results:**

Nine patients had polyneuropathy, 4 had muscle cramps, and 14 had both. Median onset of polyneuropathy and muscle cramps was 6 and 9 months after allogeneic hematopoietic stem cell transplantation, respectively. Neurophysiology revealed a predominantly axonal polyneuropathy in 20 of 26 patients. In 4 of 19 patients electromyography showed signs of myopathy or myositis. Muscle cramps were more frequent during chronic than acute GVHD and affected muscles other than calves in 15 of 18 patients. They typically occurred daily, lasted 1 to 10 minutes with medium to severe pain intensity, compromised daily activity or sleep in 12, and were refractory to therapy in 4 patients. Muscle cramps were less likely with tacrolimus treatment and signs of severe polyneuropathy, but more likely with myopathic changes in electromyography and with incipient demyelinating polyneuropathy, shown by increased high frequency attenuation of the tibial nerve. Serological studies revealed antinuclear or antimitochondrial antibodies in a subset of patients. Two of 16 patients had a serum reactivity against peripheral nervous tissue.

**Conclusion:**

Muscle cramps are associated with chronic GVHD, often compromise daily activity, and correlate negatively with axonal polyneuropathy and positively with myopathy and incipient demyelination.

## Introduction

Graft-versus-host disease (GVHD) is a frequent complication and the leading cause of morbidity after allogeneic hematopoietic stem cell transplantation (allo-HSCT) [1], [2]. It is characterized by immune-mediated multisystemic inflammation. The pathogenesis of GVHD involves proliferation and activation of allo- and autoreactive T and B lymphocytes, inappropriate generation of central and peripheral tolerance and various unspecific mechanisms of chronic inflammation [3]. While acute GVHD occurs within the first months after transplantation, chronic GVHD, whether after acute or *de novo*, may last many years and requires long-term immunosuppression [4], [5].

The classical targets of acute GVHD are skin, intestinal tract, and liver. Chronic GVHD may involve additional organs (eye, oral mucosa, lung, fascia, and genital tract) and mimic autoimmune diseases like myasthenia gravis or autoimmune cytopenias.

Neurological manifestations of GVHD have a major impact on the disease course and the quality of life [6]. They affect the peripheral more often than the central nervous system, starting usually several months to years after allo-HSCT. GVHD involves nerve roots, peripheral nerves, neuromuscular junction, or muscles. Polymyositis was reported as the most common neurologic complication of GVHD with an incidence of 2–3% after allo-HSCT, whereas immune neuropathies and myasthenia gravis occur in less than 1% [7], [8]. The diagnosis of GVHD-associated disorders of the peripheral nervous system requires exclusion of other, more frequent causes, e.g. steroid myopathy, drug-induced toxicity, or opportunistic infections [6], [9].

The clinical and electrophysiological presentation of GVHD-associated acute and chronic neuropathies is heterogeneous and resembles features of Guillain-Barré syndrome (GBS) [10], [11], chronic inflammatory demyelinating polyneuropathy (CIDP) [12], [13], or chronic immune-mediated axonal polyneuropathy [14]. Usually, the diagnostic criteria of GBS or CIDP are not met completely [15], [16], [17].

Muscle cramps are characterized by a rapid onset, painful visible or palpable contraction of single muscles or muscle groups, and residual soreness. They are typically relieved by stretching. Muscle cramps can occur in otherwise healthy persons (benign cramps), but they are also associated with a variety of pathological conditions, including lower motor neuron disorders, neuropathies, metabolic disorders, and immune-mediated mechanisms such as peripheral nerve hyperexcitability [18], [19].

Muscle cramps are a frequent, but rarely reported, complication of chronic GVHD [6], [9]. In a prospective trial evaluating physical functioning and quality of life after allo-HSCT, the incidence of daily muscle cramps was 16%, exclusively associated with moderate and severe chronic GVHD [20], [21]. It is not known, whether muscle cramps during GVHD represent an independent, immune-mediated symptom of chronic GVHD, or are related to polyneuropathy, myopathy, toxicity of long-term immunosuppression, or other secondary causes [6].

We examined peripheral nervous system complications after allo-HSCT and characterized the clinical, electrodiagnostic and laboratory features of GVHD-associated muscle cramps. We found that muscle cramps occurred in the context of chronic GVHD and were less frequent in patients with axonal neuropathy and tacrolimus treatment.

## Methods

### Patients and Clinical Outcome Measures

In this single-centre, retrospective cohort study all consecutive patients presenting to our tertiary referral centre between March 2008 and August 2010 were included if they had a history of allo-HSCT, acute or chronic GVHD, and complained of muscle cramps or other symptoms suggestive of a nervous system disorder. GVHD was diagnosed and graded according to the criteria and guidelines of the National Institutes of Health [22], [23]. All patients had typical GVHD involving at least one of the following organs: skin, eyes, oral mucosa, lungs, intestinal tract.

Exclusion criteria were: confirmed disease of the peripheral nervous system prior to allo-HSCT (particularly neuropathies, GBS, or CIDP); long-lasting (>10 years) or uncontrolled diabetes mellitus; diabetic nephropathy; other severe diseases that potentially compromise clinical or electrodiagnostic assessment of peripheral nerves; suspected GVHD of the central nervous system; and clinical findings consistent with central nervous system lesions not explained by the patient´s history.

All clinical, laboratory, and electrophysiological variables were defined prior to inclusion of patients. Standardized clinical data included: age, sex, time since allo-HSCT, origin and HLA-matching of transplant, type of GVHD (acute, chronic after acute, *de novo* chronic), immunosuppressive and neurotoxic medications, current medical and supportive therapies, laboratory data, and neurological findings, e.g. limb paresis, muscle atrophy, reflex status and vibration sense. The hemato-oncological records, including detailed data on allo-HSCT, were reviewed by a hematologist (D.W.).

Muscle cramps before and after as well as symptoms of neuropathy prior to allo-HSCT were collected according to patient’s self-report and medical records. Neuropathy after allo-HSCT was evaluated by clinical examination and electrodiagnostic studies according to the national guidelines [24]. Muscle cramps were defined as sudden-onset, painful, involuntary muscle contraction that can be relieved by passive stretching of the muscle. If available, muscle cramps were graduated by: frequency, duration, pain intensity on visual analogue scale (0 = no pain to 10 = most severe pain), localisation, and functional impairment. Response of muscle cramps to treatment was evaluated by patient’s history (n = 11) or at follow-up examinations (n = 7). Results of hematological and cerebrospinal fluid examinations (±3 months to neurological assessment), done in certified laboratories, were retrieved from the patient files.

### Electrodiagnostic Studies

The electrodiagnostic tests of multiple nerves and muscles were guided by patient’s complaints, symptoms and clinical findings. If available, follow-up or earlier post-allo-HSCT examinations were included. Electrodiagnostic studies before allo-HSCT had not been done in any patient.

Standard techniques and established laboratory normal values were used [25], [26]. The investigations comprised sensory nerve conduction studies of sural, ulnar, and median nerves and motor nerve conduction studies as well as F-wave recordings of posterior tibial, ulnar, and median nerves. Normally, we investigated nerves of the right side. Skin temperature was monitored with an infrared thermometer and was above 30°C in every case. Nerve conduction waveforms and other relevant data were stored on the recording equipment (Multiliner, Toennies Co., Höchberg, Germany). Investigated variables were: sensory nerve action potential (SNAP) amplitude, sensory nerve conduction velocity (NCV), sensory nerve distal latency, motor nerve distal compound muscle action potential (CMAP) amplitude, motor NCV, distal motor latency (DML), F-wave latency and number of A waves from motor nerves. To detect motor nerve demyelination with high sensitivity, high frequency attenuation (HFA) of the tibial nerve was used [25], [26], [27].

Standard concentric needle electromyography was done in all patients with muscle cramps or signs of polyneuropathy unless in cases of thrombopenia, disturbed coagulation or leukopenia. At least two affected muscles were examined for the presence of pathologic spontaneous activity and abnormal motor unit action potentials (MUAPs).

### Assessment of Antineuronal Antibodies

Blood samples taken between one month before to 4 months after neurological assessment, were coded for blinded investigation and tested for antineuronal antibodies at the Department of Neurology, University of Heidelberg. Using a commercial indirect immunofluorescence assay employing monkey cerebellum and unfixated peripheral nerve cryosections (Euroimmun, Lübeck, Germany) sera were analysed for anti-neuronal (anti-Hu, -Ri, -Yo, -Ma/Ta, -CV2/CRMP5, -glutamic acid decarboxylase (GAD), -amphiphysin, -N-methyl-D-aspartate receptor (NMDA-R), -α-amino-3-hydroxy-5-methyl-4-isoxazolepropionic acid receptor (AMPA-R)1 and 2, - gamma-aminobutyric acid type B1 receptor (GABA_B_R1)) and anti-glial (NMO-IgG) antibodies. Additionally, commercial recombinant cell based indirect fluorescence assays (Euroimmun, Lübeck) consisting of HEK293 cells transfected with the respective antigens and of non-transfected HEK293 cells as control substrate were used for the detection of antibodies to glutamate receptors (type NMDA, type AMPA), GABA_B_R1, glycine receptor, contactin-associated protein-2 (CASPR2), and LGI1. Briefly, microscopy slides with tissue and cell substrates were incubated with the patient’s serum diluted 1∶10, 1∶100, or 1∶320 in 1% bovine serum albumin in PBS for 1 hour. Bound human IgG was visualized by a fluorescein-conjugated goat anti-human IgG antibody (Euroimmun, Lübeck). Immunostaining patterns were evaluated independently by two experienced examiners (S.J., K.P.W.).

### Statistical Analysis

Data were analysed using SPSS version 18 (IBM, Munich, Germany). Descriptive statistics were used to describe continuous and categorical variables. Relative frequencies were computed for all variables and additionally medians and ranges for continuous variables. Longitudinal categorial data (occurrence of muscle cramps) were analysed using the McNemar test and longitudinal continuous data (electrodiagnostic tests) using the Wilcoxon-test. Correlations between occurrence of muscle cramps and other clinical and paraclinical data were obtained using Spearman´s correlation coefficient. The unpaired student´s t test was used to compare continuous data of patients with or without muscle cramps. All tests were 2-sided with an alpha level of 0.05.

### Ethics Statement

The presented study was approved by the ethics committee of the Medical Faculty of the University of Regensburg, Germany (ref 10-101-0204). As routinely collected data were used and analysed anonymously, individual consent of the patients was not requested. In a subgroup of patients, serum samples were re-analysed which had been stored previously for another study approved by the institutional ethics review board (# 02/220). Written informed consent was obtained from these patients.

## Results

### Patient Characteristics and Hematological Findings

We identified 33 patients (28 men; age 23–69 years) with nervous system complications of GVHD based on clinical and electrodiagnostic assessment. Six patients were excluded because of suspected cerebral GVHD (n = 4), adhesive spinal arachnoiditis with thoracal myelopathy (n = 1), or confirmed polyneuropathy prior to allo-HSCT (n = 1). All remaining 27 patients had a history of allo-HSCT, acute or chronic GVHD, and complained of muscle cramps or had clinical or electrodiagnostic signs of a peripheral nervous system disorder (Table 1, Table S1). Median age was 52 years (range 23–69), 25 patients were male. The most common underlying hematological malignancies were acute myeloid leukaemia (n = 13) and multiple myeloma (n = 4). Sex of donor and recipient matched in 19 and was different in 8 patients, 6 of them were male recipients with female donors. Ten transplants were derived from Human Leukocyte Antigen (HLA) matched sibling donors, 11 from matched unrelated donors, and 6 from mismatched unrelated donors. All patients had developed GVHD after allo-HSCT (acute, n = 6; chronic after acute, n = 14; *de novo* chronic, n = 7). Severity of acute GVHD was grade I in 6, grade II in 9, grade III in 4, and grade IV in 1 patient, respectively. Maximum severity of chronic GVHD was moderate in 4 and severe in 17 patients.

**Table 1:** Patients.

Characteristic	Number
Total patients	27
Sex, male	25 (93%)
Median age	52 (23–69) years
Hematologic disease	
- AML	13 (48%)
- Multiple myeloma	4 (15%)
- ALL	2 (7%)
- Hodgkin lymphoma	2 (7%)
- T cell lymphoma	2 (7%)
- CLL	1 (4%)
- CML	1 (4%)
- MDS with refractory anaemia	1 (4%)
- Non-Hodgkin-lymphoma	1 (4%)
Median duration since allo-HSCT	24 (2–106) months
Donor type	
- matched sibling	10 (37%)
- matched unrelated donor	11 (41%)
- mismatched unrelated donor	6 (22%)
GVHD type	
- Acute	6 (22%)
- Chronic quiescent onset	14 (52%)
- Chronic *de novo* onset	7 (26%)

ALL = acute lymphoblastic leukaemia, allo-HSCT = allogeneic hematopoietic stem cell transplantation, AML = acute myeloid leukaemia, CLL = chronic lymphoblastic leukaemia, CML = chronic myeloid leukaemia, GVHD = graft-versus-host disease, HLA = human leukocyte antigen, MDS = myeolodysplastic syndrome.

Every patient had received at least one therapy for the malignant disease prior to allo-HSCT (chemotherapy, n = 21; monoclonal antibodies, n = 8; autologous HSCT, n = 9; irradiation, n = 4; cytokine therapy, n = 2). Fifteen patients had one or more treatment-related comorbidities potentially involving the nervous system: present or past steroid-induced diabetes (n = 8), pathological vertebral body fractures (n = 3), leukoencephalopathy (n = 2), pressure lesion of the peronaeal nerve (n = 2), vertebral disc prolaps (n = 2), cerebral ischemia (n = 1), CMV polyradiculitis (n = 1), facial palsy and post-zoster neuralgia (n = 1), and chronic renal insufficiency (n = 1). Previous treatment of the hematological malignancy, opportunistic infections, or GVHD included at least one potentially neurotoxic therapy in all patients (cyclosporin, n = 26; tacrolimus, n = 13; spinal or total body radiation, n = 11; voriconazol, n = 10; lenalidomid, n = 4; vincristin, n = 4; linezolid, n = 3; thalidomide, n = 2; bortezomib, n = 2; cisplatin, n = 2; intrathecal methotrexate and cytarabin, n = 1; vinblastin, n = 1).

Neurologic assessment was done at a median of 24 months (range 2–106 months) after allo-HSCT. At the time of evaluation, 6 patients had acute GVHD or acute GVHD in remission, 18 patients had chronic GVHD, and 3 patients were in remission of chronic GVHD but continued to have muscle cramps which had started during GVHD. GVHD was treated with systemic or topic steroids (n = 23), mycophenolat mofetil (n = 13), tacrolimus (n = 8), cyclosporin (n = 3), everolimus (n = 3), or other immunosuppressants (n = 5).

### Neurological Findings

Twenty-three patients had symptoms and clinical signs of polyneuropathy, predominantly of a diffuse pattern, involving all limbs with proximal or distal distribution and with sensory and motor deficits (Table 2, Table S2). The median time between allo-HSCT and onset of polyneuropathy was 6 months (range 0–83). Three patients reported symptoms compatible with mild sensory polyneuropathy before allo-HSCT, but neurological or electrodiagnostic examination was not available. All 6 patients with acute GVHD had rapidly evolving polyneuropathy with moderate distal symmetric paresis (n = 4), loss (n = 4), or severe reduction (n = 2) of vibration sense, or both. Three fulfilled the clinical and electrodiagnostic criteria for axonal GBS, two patients with demyelinating polyneuropathy (one with autonomic involvement) did not fully meet the electrodiagnostic criteria for demyelinating GBS [28].

**Table 2:** Clinical findings.

Variable	Present	Median (range)
Muscle cramps		
- before allo-HSCT (n = 26)	0	
- after allo-HSCT (n = 27)	18 (67%)***	
Clinical polyneuropathy (n = 27)	23 (85%)	
Muscle cramps and polyneuropathy (n = 27)	14 (52%)	
Limb paresis, MRC ≤ 4 (n = 27)	16 (59%)	4 (2–5)
Muscle atrophy (n = 27)	11 (41%)	
Reflexes weak or absent (n = 27)		
- upper extremities	11 (41%)	
- lower extremities	23 (85%)	
Vibration sense LE impaired, ≤ 4/8 (n = 26)	18 (67%)	3 (0–8)

McNemar test (after vs. before allo-HSCT): ***p<0.001.

Eighteen patients reported muscle cramps starting at a median of 9 months (range 0–59) after allo-HSCT. No patient had muscle cramps before allo-HSCT. Fourteen patients had polyneuropathy plus muscle cramps. Only two of 6 patients with acute GVHD, but 16 of 21 patients with chronic GVHD had muscle cramps.

In clinical examination 16 of 27 patients had limb paresis (MRC≤4), 11 had symmetric distal muscle atrophy and 23 weak or absent deep tendon reflexes of the legs (Table 2). Vibration sense at the ankle was impaired (≤4/8) in 18 patients. One patient showed generalized, proximally pronounced limb weakness, bilateral ptosis and weakness of head flexors. Typical findings for myasthenia gravis, however, were absent in electrodiagnostic and serologic tests. No patient had focal or generalized myokymia or fasciculations.

### Clinical Features of Muscle Cramps

**Figure 1:**
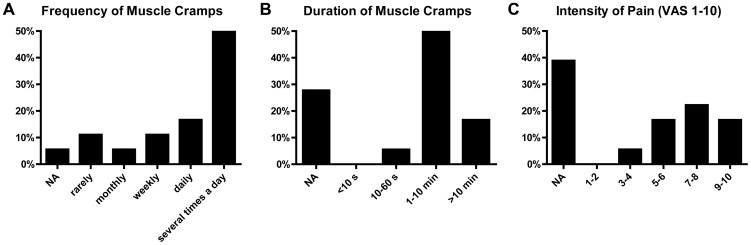
Characteristics of muscle cramps in patients with GVHD after allo-HSCT (n = 18). NA = not available; VAS = visual analogue scale.

Muscle cramps occurred at least once per day in 12 of 18 patients and lasted more than 1 minute in 9 and more than 10 minutes in 3 patients. The pain intensity was high with a median of 7–8 of 10 on a visual analogue scale (Fig. 1, Table S2). Typically muscle cramps occurred at rest and at night, but exercise-induced muscle cramps were also reported. One patient described a “warm-up” phenomenon of painful, slowly dissolving contractions of hand and forearm muscles in the morning after waking up. Calf muscles were involved in all 18 patients. In contrast to “idiopathic” muscle cramps, further muscle groups were involved in 15 patients, e.g. hamstring, hand, forearm and thoracic muscles. Twelve patients reported functional impairment by their muscle cramps and complained of severely disturbed sleep or difficulties to walk or breathe during and after the painful contractions of the affected muscles.

### Treatment of Muscle Cramps

Initial therapy of muscle cramps was magnesium in 15 of 16 patients, 10 patients had a treatment response (Table 3). Further therapies included quinine in 7 patients with 3 responders, and gabapentin, pregabalin and carbamazepine, all with minor response rates. Additionally, physical activity like muscle stretching was performed by most patients, usually being sufficient to stop the muscle cramps, except in severe forms. Muscle cramps were refractory to therapy in 4 patients. There was no difference of GVHD severity or immunosuppressive therapy between patients whose muscle cramps improved or not.

**Table 3:** Pharmacological treatment of muscle cramps.

Drug (n)	Response to treatment	
	Yes	No
Any treatment (n = 16)^a^	12	4
Magnesium (n = 15)	10	5
Quinine (n = 7)	3	4
Gabapentin (n = 3)	1	2
Pregabalin (n = 2)	1	1
Carbamazepine (n = 1)	0	1

a total 18 patients; multiple treatments per patient possible.

### Electrodiagnostic Findings

Twenty-two of 23 patients with clinical signs of polyneuropathy had abnormal findings in neurophysiology of at least one of the examined nerves. The remaining patient did not undergo nerve conduction studies but had a neurogenic pattern on electromyography. In 3 patients only sensory nerves were affected, whereas in 19 patients both sensory and motor nerves were affected. Six patients showed an exclusively axonal pattern, two patients a demyelinating pattern (tibial nerve motor NCV 30 m/s or lower), and 14 patients a pattern of neuropathy with axonal and demyelinating features (Table 4). No patient fulfilled the electrodiagnostic criteria for demyelinating GBS [28] or CIDP [29]. Some patients had severe polyneuropathy with lost SNAP of sural (n = 4), median (n = 3), or ulnar (n = 1) nerve, or lost CMAP of the tibial (n = 2) nerve. Most common abnormalities were decreased SNAP amplitude and mildly decreased sensory NCV of the median and ulnar nerves. Approximately half of the patients had absent or delayed F waves from motor nerves and a decreased motor NCV of the tibial nerve.

**Table 4:** Electrodiagnostic studies.

Variable	Abnormal	Median (range)
*Neurophysiology (n = 26)*		
Sural nerve		
- SNAP amplitude, µV (n = 26)	4 (15%)	3.4 (0–16.5)
- NCV, m/s (n = 22)	7 (32%)	43.0 (24.7–54.6)
Tibial nerve		
- DML, ms (n = 24)	5 (21%)	4.1 (2.1–6.4)
- distal CMAP amplitude, mV (n = 26)	7 (27%)	4.6 (0–19.1)
- NCV, m/s (n = 23)	10 (43%)	39.3 (29.0–44.4)
- HFA, % (n = 22)	2 (9%)	28.0 (7.0–50.0)
- F-wave latency, ms (n = 26)	11 (42%)	57.4 (52.2–67.0)
- A waves, ≥ 3 (n = 24)	6 (25%)	
Median nerve		
- SNAP amplitude, µV (n = 20)	16 (80%)	3.4 (0–21.8)
- sensory NCV, m/s (n = 17)	15 (88%)	41.0 (36.0–46.4)
- DML, ms (n = 17)	2 (12%)	3.6 (2.8–4.9)
- distal CMAP amplitude, mV (n = 17)	1 (6%)	6.6 (2.3–17.0)
- motor NCV, m/s (n = 17)	5 (29%)	50.8 (46.5–61.7)
- F wave latency, ms (n = 13)	6 (46%)	31.4 (28.8–33.8)
- A waves, ≥ 3 (n = 12)	2 (17%)	
Ulnar nerve		
- SNAP amplitude, µV (n = 20)	16 (80%)	2.9 (0–7.9)
- sensory NCV, m/s (n = 19)	9 (47%)	43.0 (32.1–52.8)
- DML, ms (n = 12)	1 (8%)	3.0 (1.9–3.5)
- distal CMAP amplitude, mV (n = 12)	1 (8%)	8.9 (1.0–14.8)
- motor NCV, m/s (n = 12)	4 (33%)	51.4 (43.6–56.3)
- F wave latency, ms (n = 7)	3 (43%)	31.0 (28.6–33.6)
*Electromyography (n = 19)*		
Muscles affected by cramps or paresis or atrophy		
- neurogenic MUAPs	10 (53%)	
- small MUAPs	4 (21%)	
- fibrillation potentials	3 (16%)	
- no pathology	5 (26%)	

CMAP = compound muscle action potential, DML = distal motor latency, HFA = high frequency attenuation, MUAP = motor unit action potential, NCV = nerve conduction velocity, SNAP = sensory nerve action potential.

Nineteen patients underwent electromyography of muscles affected by cramps, pareses, or both (Table 4). In 10 patients MUAPs had a neurogenic pattern, in one patient pathologic spontaneous activity and small amplitudes as signs of myositis were found, and 3 patients had a myopathic pattern with small amplitudes (assumed steroid myopathy because of lacking fibrillation potentials and normal serum creatine kinase levels). All patients with small MUAPs had frequent muscle cramps and two had a concomitant polyneuropathy. Since no patient showed muscle cramps at examination in our outpatients department, electromyography could not be done during a cramp episode. In between muscle cramps no “myotonia-like” patterns, doublets, and repetitive or complex repetitive discharges were found in affected muscles. Seven of 10 patients with a neurogenic electromyography had moderate to severe limb paresis and muscular atrophy, all had weak or absent deep tendon reflexes. Nerve conduction studies confirmed polyneuropathy in 9 of these patients. All 4 patients without clinical and electrodiagnostic signs of polyneuropathy had muscle cramps.

In summary, the electrodiagnostic profile of most patients (n = 20) was consistent with predominantly axonal neuropathy.

### Laboratory Findings

Cerebrospinal fluid (CSF) examined in 6 patients was abnormal in 5 (2 acute, 3 chronic GVHD; Table 5). Mild pleocytosis with a lymphocytic infiltrate was found in two patients, no infectious cause was identified. Five patients had increased CSF protein of >450 mg/l, 4 of them with compromised blood-CSF-barrier. No oligoclonal bands or intrathecal immunoglobulin (Ig) synthesis were found. All 5 patients with increased CSF protein had a polyneuropathy and 3 of them additionally muscle cramps.

**Table 5:** Cerebrospinal fluid findings.

Variable	Present	Median (range)
Cerebrospinal fluid (n = 6)		
- increased leukocyte count, >5/µl	2 (33%)	2 (1–46)
- increased protein, >450 mg/l	5 (83%)	701 (430–1850)
- increased lactate, >2.1 mmol/l (n = 5)	3 (60%)	2.3 (1.7–4.3)
- intrathecal IgG synthesis^a^ (n = 5)	0	
- compromised blood-CSF-barrier^a^ (n = 5)	4 (80%)	

a assessed by calculation of the CSF/serum quotient [43].

CSF = cerebrospinal fluid, IgG = immunoglobulin G.

Serum creatine kinase was elevated in 1 of 13 patients (median 74 U/l, range 36–1052 U/l), lactate dehydrogenase was increased in 14 of 23 patients (median 256 U/l, range 174–677 U/l), platelets were abnormal in 8 of 25 patients (<100.000/µl in 7, >400.000/µl in 1, median 194.000/µl, range 24.000–454.000/µl). Four patients with multiple myeloma had an IgG paraprotein, 3 of them had a *de novo* polyneuropathy and all had muscle cramps, both occurring after allo-HSCT in the course of GVHD.

### Serological Studies

Antineuronal and anti-ion-channel antibodies have been described in some patients with peripheral nerve hyperexcitability and muscle cramps [19]. Hence, a comprehensive set of antibodies was tested, but was negative in all patients (n = 16, 10 with muscle cramps; Table S3). In particular, no reactivity against the voltage-gated potassium channel associated proteins leucine-rich, glioma inactivated 1 protein (LG1) and contactin-associated protein-2 (CASPR2) [30] was found. Using an indirect immunofluorescence assay with monkey central and peripheral nervous tissue, however, serum reactivity against peripheral nerve myelin was found in two patients. One had severe muscle cramps several times a day with long duration and high pain intensity as well as mild polyneuropathy with areflexia and a CSF protein of 1676 mg/l (patient #24); moreover, he had a high titer (1∶3200) of serum antinuclear antibodies reactive to the mitotic spindle apparatus. The second patient (patient #13) did not have muscle cramps, but severe axonal polyneuropathy, unable to elevate limbs against gravity. In another patient (patient #16), serum antinuclear antibodies (titer of 1∶160; Hep2 cell-based assay) were positive in sections of cerebellum and plexus myentericus. Further patients also had antinuclear antibodies or antimitochondrial antibodies reactive to nervous tissue, however, confirmation assays with Hep2 cell-based assays were either negative or not done.

### Follow-up Examinations

Ten patients were seen again (last follow-up after 1 to 27 months, median 11 months), one other patient was added in the longitudinal analysis, who had undergone electrodiagnostic assessment after allo-HSCT and 102 months prior to the current examination. Having received a combination of various symptomatic and immunosuppressive therapies 4 of 7 patients reported an improvement and 3 reported no change or deterioration of muscle cramps. Clinically, paresis as well as sensory deficits improved in 3, were stable in 5, and worsened in 3 patients.

**Figure 2:**
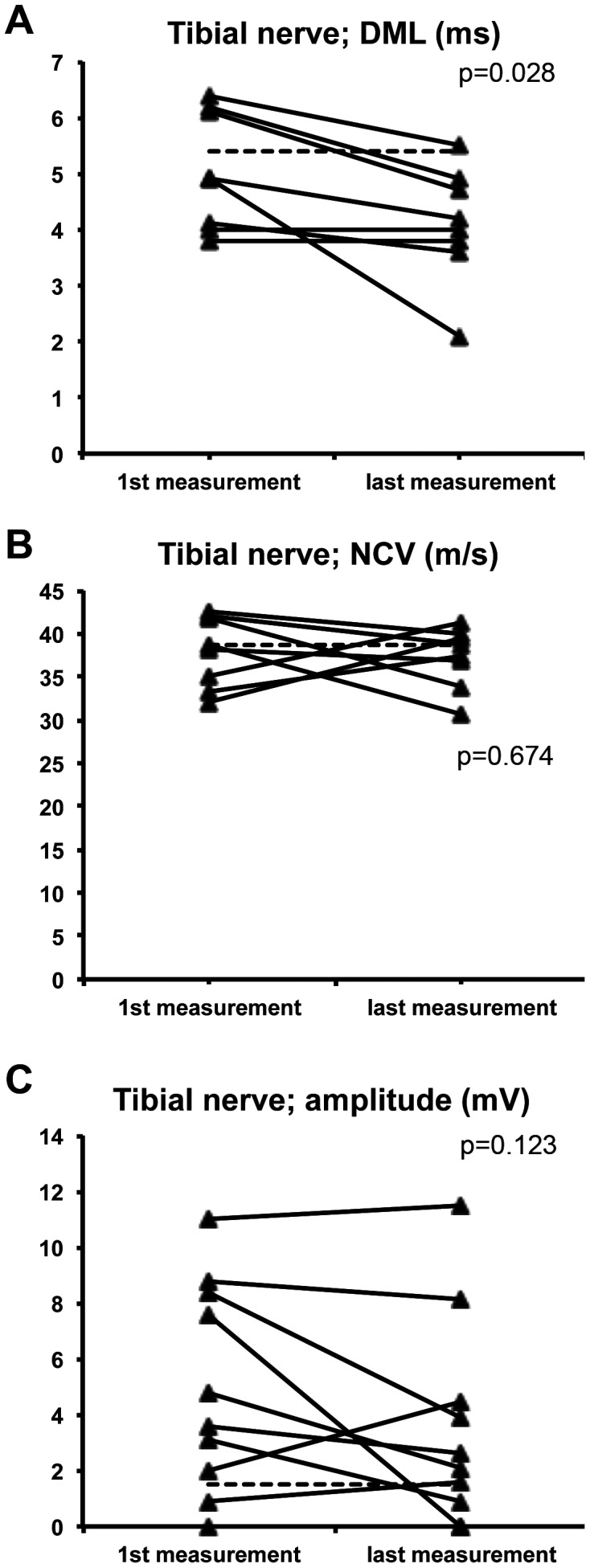
Longitudinal follow-up of neurography of the tibial nerve. The dotted line depicts the normal value. Results of a Wilcoxon test (first versus last examination) are shown. DML = distal motor latency, NCV = nerve conduction velocity.

There was a significant improvement of distal motor latency of the tibial nerve (n = 8; mean 5.1 ms versus 4.0 ms; p = 0.028), whereas NCV (n = 8; mean 38.0 m/s versus 37.3 m/s) and amplitude (n = 10; mean 5.0 mV versus 3.5 mV) were unchanged, the latter with a trend towards deterioration (Fig. 2). No significant changes were seen in electrodiagnostic examinations of the sural (n = 10), ulnar (n = 6), and median (n = 5) nerve.

Hematological follow-up for at least 12 months was available in all patients. Twelve patients died, 7 due to GVHD or treatment-related opportunistic infections, 4 to exacerbation of GVHD, and one to recurrence of the hematological malignancy.

### Variables Associated with Muscle Cramps

**Figure 3:**
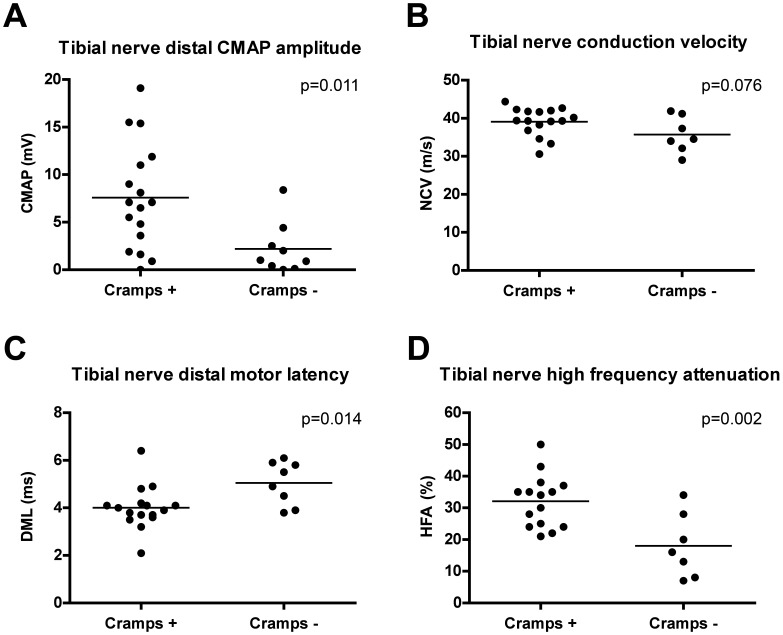
Neurophysiological assessment of polyneuropathy in patients with or without muscle cramps (Cramps+/Cramps−). Results of an unpaired, two-tailed *t* Test are shown. CMAP = compound muscle action potential, DML = distal motor latency, HFA = high frequency attenuation, NCV = nerve conduction velocity.

To evaluate the association between the occurrence of muscle cramps and clinical, electrodiagnostic, and laboratory variables, multiple regression analysis was done (Table 6). Muscle cramps correlated positively with the time since allo-HSCT and negatively with female sex of recipient and prior or present use of tacrolimus. Female gender of donor in male recipients and the presence of chronic GVHD seemed to be associated with muscle cramps, statistical significance, however, was not reached. Many signs of axonal neuropathy correlated negatively with the occurrence of muscle cramps (clinical: low muscle strength, presence of muscle atrophy; electrophysiological: high DML, low distal CMAP amplitude, neurogenic MUAPs on electromyography). Patients with muscle cramps had significantly higher distal CMAP amplitudes (7.6 mV versus 2.2 mV; p = 0.011), lower distal motor latency (4.0 ms versus 5.1 ms; p = 0.014), and higher conduction velocity (39.1 m/s versus 35.7 m/s; p = 0.076) in the tibial nerve (Fig. 3A–C). Small MUAPs correlated with the presence of muscle cramps, but only 1 patient showed electromyographic signs of myositis. Furthermore, high frequency attenuation (HFA), a marker of incipient myelin damage [25], [27], was significantly correlated with the occurrence of muscle cramps; patients with muscle cramps had a significantly higher HFA than patients without muscle cramps (32.1 versus 18.0; p = 0.002, Fig. 3D). Neither CSF and other laboratory variables nor severity of GVHD or localized sclerosis, an antibody-mediated manifestation of chronic GVHD, correlated with the occurrence of muscle cramps. Prior or present use of tacrolimus significantly correlated with the absence of muscle cramps.

**Table 6:** Multiple regression analysis to detect variables associated with muscle cramps.

Variable	r	*p* value
Medical history		
- Time since allo-HSCT	**0.474**	**0.012**
- HLA-matching	0.057	0.776
- Female sex of donor^a^	0.378	0.052
- Female sex of recipient	**−0.400**	**0.039**
- Acute GVHD	**−**0.239	0.230
- Chronic GVHD	0.378	0.052
- Cyclosporin ever	0.277	0.161
- Tacrolimus ever	**−0.577**	**0.002**
Clinical findings		
- Clinical PNP	**−**0.295	0.135
- Muscle strength, MRC	**0.540**	**0.004**
- Muscle atrophy	**−0.533**	**0.004**
- weak or absent reflexes LE	**−**0.295	0.135
- Vibration sense	0.149	0.459
Neurophysiology (tibial nerve)		
- DML	**−0.480**	**0.018**
- Distal CMAP amplitude	**0.501**	**0.009**
- NCV	0.356	0.095
- HFA	**0.609**	**0.003**
- F-wave latency	**−**0.360	0.143
Electromyography		
- fibrillation potentials	0.100	0,693
- neurogenic MUAPs	**−0.800**	**0.000**
- small MUAPs	**0.478**	**0.045**

a male recipients only.

Shown is the Spearman correlation coefficient. Dependent variable: presence of muscle cramps. Bold values depict statistically significant correlations. CMAP = compound muscle action potential, DML = distal motor latency, HFA = high frequency attenuation, LE = lower extremity, MRC = Medical research council, MUAP = motor unit action potential, NCV = nerve conduction velocity, SNAP = sensory nerve action potential.

Hence, we conclude that muscle cramps in GVHD patients are less frequent in patients with axonal neuropathy and tacrolimus treatment. On the other hand, they seem to be associated with incipient demyelinating polyneuropathy and myopathy, even in the absence of myositis.

## Discussion

In this study, we evaluated peripheral nervous system disorders associated with GVHD after allo-HSCT. Axonal neuropathy and muscle cramps were the major complaints; their occurrence was negatively correlated to each other.

Affecting as many as 64% of long-term survivors, neurological complications are common after allo-HSCT [31]. They are associated with long-term immunosuppression and GVHD. We confirm that disorders of the peripheral nervous system have a delayed onset and usually occur in the setting of chronic GVHD [7], [16], [17], [32]. Whereas all patients with peripheral nervous system complications in our study per inclusion criteria had acute or chronic GVHD, others have reported the occurrence of GBS, CIDP, brachial plexopathy, or multiple radiculopathies after allo-HSCT in the absence of preceding classical GVHD [10], [11], [33]. Albeit uncommon, it is conceivable that GVHD may manifest first in the peripheral nervous system.

We have identified 27 patients who presented with peripheral nervous system complications (neuropathies, muscle cramps, or myopathy) during GVHD after allo-HSCT. Although our study was not designed to identify the incidence of these complications, GVHD-associated neuropathies seem to be more frequent than reported previously (1%) [7], [8]. Of ∼200 long term survivors treated at our transplant centre for GVHD within the observation period, 23 had polyneuropathy and 18 muscle cramps. These numbers are likely to be underestimated, since no vigorous search strategy was applied. As only one patient had myositis and no patient had myasthenia gravis, our data suggest that neuropathies and muscle cramps are more frequent during GVHD than other peripheral nervous system complications. Few previous studies used electrodiagnostic tests to identify patients with peripheral nervous system complications. Padovan *et al.* reported that 12 of 58 (21%) patients developed a *de novo* polyneuropathy after allo-HSCT and 14 (24%) distal muscular atrophy, associated with preceding severe acute GVHD, metabolic disturbances and prolonged immunosuppression [34]. In our study only two patients had a demyelinating pattern of neuropathy. The majority had an axonal or unclassified pattern, affecting sensory nerves more often than motor nerves. Although 5 of 6 patients had protein elevation in the CSF, none fulfilled the electrodiagnostic criteria for demyelinating GBS or CIDP.

Little is known about the etiology of GVHD-associated neuropathies. Often, they manifest as GBS- or CIDP-like disease (reviewed in [6], [7]), or as mild progressive neuropathy [16], [34]. The close temporal relationship with the occurrence of GVHD and improvement upon immunosuppression in some patients suggest that neuropathies indeed are part of GVHD. As such, similar to idiopathic immune-mediated neuropathies, the generation of clonally expanded T cells [35], [36] or of a humoral immune response against peripheral nervous tissue [37] could be causative. In our patients, no immunological studies or histology were done. Others have reported vasculitic changes [38], an infiltration of T cells and deposits of immunoglobulins [35], or a loss of myelinated fibres with perineural fibrosis [39] in sural nerve biopsies. When evaluating neuropathies in patients with GVHD after allo-HSCT, multiple causes related to the therapy or the hematological disorder have to be considered [6], [7], [8]. None of our patients had obvious critical-illness neuropathy or acute neurotoxicity due to high-dose chemotherapy and all patients were in remission of their hematological disease. However, we cannot exclude that steroid-induced diabetes and prior or present use of neurotoxic drugs, e.g. cyclosporin, voriconazol, or lenalidomid contributed to the neuropathies. The heterogenous presentation of GVHD-associated neuropathies might be due to different etiologies involving immune-dependent and -independent causes and reflect the fact that no common diagnostic criteria have been specified so far.

Muscle cramps have been reported only very occasionally after allo-HSCT [21]. Greenspan *et al.* [40] described a patient with severe muscle cramps as late complication of chronic GVHD, associated with primary sensory polyneuropathy; they observed an improvement after immunosuppressive treatment with steroids and azathioprine. In our study muscle cramps were more frequent during chronic than acute GVHD, had a high frequency and pain intensity, and compromised daily activity or sleep in most patients. The majority of muscle cramps improved upon physical measures (stretching) and symptomatic pharmacological treatment. All patients additionally received immunosuppression for GVHD. Owing to the retrospective design of the study, no formal claims regarding the efficacy of treatment can be made. Nevertheless, the clinical course with improving neuropathy and muscle cramps during immunosuppressive treatment in some patients described here and elsewhere [40] indicates an immunological mediated etiology. Given the wide array of immunosuppressive drugs used for GVHD, drug-induced peripheral nerve toxicity is a possible contributing mechanism, although tacrolimus was inversely associated with muscle cramps. Since all but one patient had received cyclosporin, no conclusion can be drawn whether this is a tacrolimus specific effect or generally caused by calcineurin inhibitors. All these results might be biased by the relatively small numbers of patients and should be corroborated by larger prospective studies.

The anatomic site of muscle cramp generation still is a matter of debate, ranging from abnormal discharges of anterior horn cells, ephaptic transmission in the injured peripheral nerve, to aberrant excitation of motor nerve terminals [18], [41]. Unfortunately, we did not manage to record electromyography during a cramp episode. Thus, we cannot formally prove the peripheral nervous origin of the muscle cramps in our patients. We found, however, a significant correlation between the occurrence of muscle cramps and subtle demyelination, shown by increased HFA. Our observation raises the possibility that muscle cramps and subclinical demyelinating damage are early signs of neuropathy and precede severe axonal damage in GVHD-associated neuropathies. With more pronounced axonal neuropathy, muscle cramps may even improve. The pathophysiological basis of this observation is unknown, but it might be speculated that a humoral or cellular immune response to peripheral myelin in the context of chronic GVHD is causative. We could exclude the presence of voltage-gated potassium channel antibodies which previously were associated with peripheral nerve hyperexcitability [19], [30], but found a hitherto unspecified serum reactivity against peripheral myelin in two of the patients.

Our study population had a remarkable male preponderance of >90%. The retrospective design makes selection bias possible. In a report of 28 post-allo-HSCT patients, however, 74% with a GBS-like manifestation and 89% with CIDP were male [17]. Female donor to male recipient allo-HSCTs are associated with a higher rate of chronic GVHD, since the donor immune system may target host H-Y antigens by H-Y-reactive T cells and antibodies [42]. Therefore, H-Y antigens serving as *de novo* antigens for female donors may be targeted within neuronal structures leading to damage of the peripheral nervous system.

This study has several implications for the management of GVHD patients. First, baseline examinations before allo-HSCT seem useful for assessment of subsequent complications of the peripheral nervous system and should be established in clinical routine. Second, physicians in care of patients with allo-HSCT should recognize signs and symptoms of neuropathy and peripheral nerve hyperexcitability and refer patients for neurological examination and electrodiagnostic testing. Third, muscle cramps often have a high frequency and intensity resulting in functional impairment. Therefore, prompt physical and symptomatic therapy is warranted. After exclusion of other causes of muscle cramps, immunosuppressive therapy may be changed or increased, at least in patients with further systemic signs of active chronic GVHD.

In summary, we describe muscle cramps affecting patients with GVHD after allo-HSCT as a new and clinically relevant entity. We showed that muscle cramps occur less frequently in patients with severe polyneuropathy or tacrolimus treatment; they are more likely with myopathic changes in electromyography and incipient demyelination. Further prospective studies are warranted to characterize the immunopathogenesis and best treatment of peripheral nervous system disorders after allo-HSCT.

## Supporting Information

Table S1 Hematology.
(PDF)

Table S2 Peripheral nervous system complications.
(PDF)

Table S3 Anti-neuronal antibodies.
(PDF)
