# Supplementary material for: Muscle Cramps and Neuropathies in Patients with Allogeneic Hematopoietic Stem Cell Transplantation and Graft-versus-Host Disease
Source: PLoS One. 2012 Sep 17;7(9):e44922. doi: 10.1371/journal.pone.0044922 (PMC3444502; doi:10.1371/journal.pone.0044922)
Supplement: Table S1 — Hematology. (PDF) [file pone.0044922.s001.pdf]

Table S1: Hematology

Pat. #	Sex	Age (yrs)	Underlying Malignancy	HLA-Match	Sex of Donor	Related Donor	Number of allo-HSCTs	aGVHD Organ Involvement and Overall Grade	Day Onset aGVHD[a]	cGVHD Organ Involvement and Maximum Degree	Day Onset cGVHD[a]	Maximum Severity of cGVHD	Localized Sclerosis (Skin, Lung)	Immunosuppressive Treatment for cGVHD	TAC ever	CsA ever	Day Neurological Evaluation[a]	Current Severity of GVHD[b]	Current Immunosuppression[b]
1	m	52	CLL	N	m	N	1	Skin, GIT (IV°)	16	Skin (III°), eye (III°)	88[c]	Severe	N	Pred, TAC, ECP, Campath	Y	N	117	Moderate	TAC, Pred
2	m	68	AML	Y	m	N	1	Skin (II°)	23	Skin (III°), GIT (II°)	315	Severe	N	Pred, MMF, ECP	N	Y	759	None[d]	MMF, Pred
3	m	57	MM	Y	f	Y	1	None		Skin (III°), fascia (III°), lung (II°), eye (I°), liver (I°), oral (I°)	365	Severe	Y	Pred, MMF, CsA, Imatinib, ECP	N	Y	571	Severe	CsA, MMF, Pred, Imatinib
4	m	55	CML	N	m	N	1	Skin (II°)	10	Skin (II°), GIT (II°)	253	Moderate	N	Pred, CsA, MMF	N	Y	3237	None[d]	None
5	m	51	HL	Y	m	Y	1	None		Skin (III°), GIT (II°), fascia (II°)	1078	Severe	Y	Pred, MMF, Everolimus, ECP, Rituximab	N	Y	2483	Severe	Everolimus, MMF, Pred
6	m	60	MM	Y	m	N	1	Skin (II°)	11	Lung (III°), eye (II°), oral (I°)	446	Severe	N	Pred, MMF	N	Y	1734	Severe	Pred
7	m	61	MM	Y	f	Y	1	Skin (II°)	25	Skin (III°), GIT (II°)	368	Severe	N	ECP, Pred, MMF, CsA	N	Y	715	Moderate[e]	Pred, MMF
8	m	67	MDS/AML	Y	m	N	1	GIT (II°)	50	Lung (II°), eye (II°), oral (I°)	302	Severe	N	Pred, MMF, Everolimus	N	Y	665	Severe	Pred, MMF, Everolimus
9	m	52	ALL	Y	m	N	1	Skin, GIT (III°)	114	Skin (III°)	351	Severe	Y	Pred, Everolimus, MMF	N	Y	1066	Moderate	MMF, Pred
10	m	45	AML	Y	m	N	1	None		Skin (III°), fascia (III°), polyserositis (III°), eye (II°), oral (I°)	264	Severe	Y	Pred, ECP, TAC, Everolimus, Imatinib, Etanercept	Y	Y	770	Severe	MMF, Pred, Everolimus, Etanercept
11	m	29	ALL	Y	m	N	1	Skin (I°)	21	None			N		N	Y	83	Mild	Pred, CsA
12	m	48	MDS/AML	Y	m	N	1	Skin (I°)	20	Skin (II°)	248	Moderate	N	TAC, Pred	Y	Y	91	Mild	TAC
13	m	60	MDS-RA	Y	m	Y	1	Skin (I°)	62	Skin (III°), oral (I°), liver (I°)	160	Severe	N	Pred, MMF, Rituximab, ECP	Y	Y	544	Severe[e]	MMF, Pred, ECP
14	m	23	AML	Y	f	Y	1	Skin, liver, GIT (III°)	110	Skin (III°), lung (III°), upper GIT (II°), eye (II°)	270	Severe	Y	MMF, TAC, CsA, Rituximab, Imtinib, ECP, Sirolimus, PUVA	Y	Y	2165	Severe	Pred, Imatinib, MMF
15	m	51	fNHL	N	m	N	1	Skin (III°)	14	None			N		Y	Y	156	Severe	Pred
16	m	41	AML	Y	m;m;f	N	3	None		Lung (III°), liver (II°), eye (II°), oral (I°), fascia (I°)	270	Severe	N	Pred, CsA, MMF, Everolimus,	N	Y	1410	Severe	Pred, MMF
17	f	40	MDS/AML	N	m	N	1	Skin (I°)	11	None			N		N	Y	126	Moderate[e]	CsA
18	f	30	HL	Y	m	Y	1	Skin (I°)	20	Lung (III°)	469	Severe	N	Pred, CsA, TAC Everolimus, ECP, MMF, Imatinib	Y	Y	911	Severe	TAC, MFM, Pred
19	m	66	MDS/AML	N	m	N	1	Skin (II°)	10	None			N		Y	Y	34	Moderate	TAC, MFM, Pred
20	m	48	AML	Y	m	N	1	Skin, GIT (II°)	15	None			N		Y	Y	55	Moderate	TAC, Pred
21	m	58	MM	Y	m;m	Y	2	None		Skin (III°)	938	Severe	Y	None (localized scleroderma)	N	Y	1058	Mild	Pred
22	m	48	AML	Y	m	Y	1	Skin (I°)	20	Eye (III°), oral (I°), lung (I°)	196	Severe	N	Pred	N	Y	304	Moderate	Pred
23	m	58	TCL	Y	f	Y	1	Skin (II°)	36	Skin (II°), eye (I°), oral (I°)	173	Moderate	N	Pred, CsA, MMF	N	Y	2637	None[d]	None
24	m	52	AML	N	m	N	1	None		Skin (II°)	180	Moderate	N	TAC, Pred	Y	Y	195	Moderate	TAC, Pred
25	m	64	AML	Y	m	N	1	GIT (III°)	220	Skin (III°), eye (II°)	434	Severe	N	Pred, MMF	Y	Y	672	Mild	Pred, MMF
26	m	37	TCL	Y	f	Y	1	None		Skin (III°), eye (II°), genital (II°), fascia (II°), oral (I°), lung (I°)	258	Severe	Y	Pred, TAC, Everolimus, ECP, MMF	Y	Y	1122	Severe	TAC, Pred, MTX
27	m	69	MDS/AML	Y	m	N	1	Skin (II°)	34	None			N		Y	Y	51	Moderate[e]	TAC, Pred, MTX

aGVHD = acute graft-versus-host disease; ALL = acute lymphoblastic leukaemia; allo-HSCT = allogeneic hematopoietic stem cell transplantation; AML= acute myeloid leukaemia; cGVHD = chronic graft-versus-host disease; CML = chronic myeloid leukaemia; CLL = chronic lymphoblastic leukaemia; CsA = Cyclosporine A; ECP = extracorporeal photopheresis; f = female; fNHL= folicular non-Hodgkin lymphoma; GIT = gastrointestinal tract; HL = Hodgkin lymphoma; HLA = human leukocyte antigen; m = male; MDS/AML= acute myeloid leukaemia evolving from myeolodysplastic syndrome; MDS-RA = MDS with refractory anaemia; MFM = mycophenolatmofetil; MM = multiple myeloma; N = no; NA = not available; Pred = prednisolone; PUVA = psoralen and ultraviolet A; TAC = Tacrolimus; TCL = T-cell-lymphoma; Y = yes.

[a] relative to allo-HSCT (days)
[b] at date of first neurological examination
[c] progressive onset
[d] clinical symptoms of GVHD resolved, but muscle cramps initially occurring during GVHD persisted
[e] if GBS/polyneuropathy are considered as a manifestations of GVHD; all other manifestations were in complete remission
