# Supplementary material for: Muscle Cramps and Neuropathies in Patients with Allogeneic Hematopoietic Stem Cell Transplantation and Graft-versus-Host Disease
Source: PLoS One. 2012 Sep 17;7(9):e44922. doi: 10.1371/journal.pone.0044922 (PMC3444502; doi:10.1371/journal.pone.0044922)
Supplement: Table S2 — Peripheral nervous system complications. (PDF) [file pone.0044922.s002.pdf]

Table S2: Peripheral nervous system complications

Pat. #	Sex	Age (yrs)	Clinical and/or Electrodiagnostic Signs of			Features of Muscle Cramps						
			PNP	Myositis or Myopathy	Muscle Cramps	allo-HSCT to Onset	Frequency	Duration	Intensity (VAS)	Localisation	Functional Disability	Therapy (Response Yes/No)
1	m	52	Y	N	N							
2	m	68	Y	NA	Y	12 months	NA	NA	NA	GM + other muscles	N	Mg (Y)
3	m	57	Y	Y	Y	16 months	> once a day	1-10 min	NA	GM + other muscles	Y	Quinine (Y)
4	m	55	Y	NA	Y	<1 month	weekly	1-10 min	6	GM + other muscles	Y	Mg (Y)
5	m	51	Y	N	Y	59 months	> once a day	1-10 min	NA	GM + other muscles	Y	Mg (N); Quinine (N)
6	m	60	Y	NA	Y	NA	daily	NA	NA	GM + other muscles	Y	Mg (N); GBP (N)
7	m	61	Y	NA	Y	NA	monthly	NA	6	GM + other muscles	N	No therapy yet
8	m	67	N	N	Y	23 months	> once a day	> 10 min	9	GM + other muscles	Y	Mg (Y); Quinine (Y)
9	m	52	Y	N	Y	27 months	> once a day	NA	4	GM + other muscles	Y	Mg (Y)
10	m	45	Y	N	N							
11	m	29	Y[a]	N	Y	<1 month	> once a day	1-10 min	5	GM + other muscles	Y	Mg (N); Quinine (Y)
12	m	48	N	N	Y	1 month	> once a day	1-10 min	9	GM only	Y	Mg (N); Quinine (N)
13	m	60	Y	N	N							
14	m	23	N	Y	Y	28 months	daily	10-60 sec	7	GM + other muscles	N	No therapy yet
15	m	51	Y[a]	NA	Y	NA	rare	NA	NA	GM only	N	Mg (Y)
16	m	41	Y	Y	Y	NA	daily	> 10 min	9	GM + other muscles	Y	Mg (Y); GBP (N); PGB (Y)
17	f	40	Y[a]	N	N							
18	f	30	Y	N	N							
19	m	66	Y[b]	N	N							
20	m	48	Y	NA	N							
21	m	58	N	Y	Y	NA	> once a day	1-10 min	NA	GM + other muscles	N	Mg (Y); Quinine (N)
22	m	48	Y	N	Y	9 months	rare	> 10 min	NA	GM + other muscles	N	Mg (Y); GBP (Y)
23	m	58	Y	NA	Y	1 month	weekly	1-10 min	7	GM + other muscles	Y	Mg (Y); PGB (N); CBZ (N)
24	m	52	Y	NA	Y	6 months	> once a day	1-10 min	8	GM only	Y	Mg (N)
25	m	64	Y	N	N							
26	m	37	Y	N	Y	6 months	> once a day	1-10 min	7	GM + other muscles	Y	Mg (Y); Quinine (N)
27	m	69	Y[c]	N	N							

allo-HSCT = allogeneic hematopoietic stem cell transplantation; CBZ = carbamazepin; f = female; GBP = gabapentin; GM = gastrocnemius muscle; m = male; Mg = magnesium salts; min = minutes; N = no; NA = not available; PGB = pregabalin; PNP = polyneuropathy; sec = seconds; VAS = visual analogue scale (0-10); Y = yes.

[a] clinically and neurophysiologically axonal GBS
[b] clinically GBS (primarily demyelinating, however electrodiagnostic criteria for demyelinating GBS not met)
[c] clinically GBS with autonomic failure (primarily demyelinating, however electrodiagnostic criteria for demyelinating GBS not met)
