# Supplementary material for: Muscle Cramps and Neuropathies in Patients with Allogeneic Hematopoietic Stem Cell Transplantation and Graft-versus-Host Disease
Source: PLoS One. 2012 Sep 17;7(9):e44922. doi: 10.1371/journal.pone.0044922 (PMC3444502; doi:10.1371/journal.pone.0044922)
Supplement: Table S3 — Anti-neuronal antibodies. (PDF) [file pone.0044922.s003.pdf]

Table S3: Anti-neuronal antibodies

<table>
<tr><th>Pat. #</th><th>Peripheral nerve[a]</th><th>Cerebellum[b]</th><th>Intestine[c]</th><th>Hu</th><th>Ri</th><th>Yo</th><th>Ma/Ta</th><th>CV2</th><th>GAD</th><th>Amphi-physin</th><th>NMDA-R</th><th>AMPA-R1</th><th>AMPA-R2</th><th>GABA$_B$R1</th><th>LG1</th><th>CASPR2</th><th>NMO-IgG</th></tr>
<tr><td>1</td><td>neg.</td><td>neg.</td><td>neg.</td><td rowspan="16">neg.</td><td rowspan="16">neg.</td><td rowspan="16">neg.</td><td rowspan="16">neg.</td><td rowspan="16">neg.</td><td rowspan="16">neg.</td><td rowspan="16">neg.</td><td rowspan="16">neg.</td><td rowspan="16">neg.</td><td rowspan="16">neg.</td><td rowspan="16">neg.</td><td rowspan="16">neg.</td><td rowspan="16">neg.</td><td rowspan="16">neg.</td></tr>
<tr><td>2</td><td>neg.</td><td>neg.</td><td>neg.</td></tr>
<tr><td>5</td><td>neg.</td><td>neg.</td><td>neg.</td></tr>
<tr><td>8</td><td>neg.</td><td>neg.</td><td>neg.</td></tr>
<tr><td>11</td><td>neg.</td><td>neg.</td><td>neg.</td></tr>
<tr><td>12</td><td>neg.</td><td>ANA</td><td>neg.</td></tr>
<tr><td>13</td><td>myelin</td><td>ANA</td><td>neg.</td></tr>
<tr><td>15</td><td>neg.</td><td>AMA</td><td>neg.</td></tr>
<tr><td>16</td><td>neg.</td><td>ANA</td><td>ANA</td></tr>
<tr><td>17</td><td>neg.</td><td>neg.</td><td>neg.</td></tr>
<tr><td>19</td><td>neg.</td><td>neg.</td><td>neg.</td></tr>
<tr><td>20</td><td>neg.</td><td>neg.</td><td>neg.</td></tr>
<tr><td>22</td><td>neg.</td><td>neg.</td><td>neg.</td></tr>
<tr><td>24</td><td>myelin</td><td>neg.</td><td>neg.</td></tr>
<tr><td>26</td><td>neg.</td><td>ANA</td><td>ANA</td></tr>
<tr><td>27</td><td>neg.</td><td>neg.</td><td>neg.</td></tr>
</table>

AMA = antimitochondrial antibodies; AMPA-R = amino-3-hydroxy-5-methyl-4-isoxazolepropionic acid receptor; ANA = antinuclear antibodies; CASPR2 = contactin-associated protein-2; GABA$_B$R1 = gamma-aminobutyric acid type B1 receptor; GAD = glutamic acid decarboxylase; LG1 = leucine-rich glioma inactivated 1 protein; neg. = negative; NMDA-R = N-methyl-D-aspartate receptor.

[a] 1:320 dilution of serum
[b] 1:10 dilution of serum
[c] 1:10 dilution of serum; plexus myentericus
